# Supplementary material for: Single-Ion Anisotropy-Stabilized Short-Period Helimagnetism in Frustrated Chiral Co5TeO8
Source: Research (Wash D C). 2026 Jun 25;9:1334. doi: 10.34133/research.1334 (PMC13294545; doi:10.34133/research.1334)
Supplement: Supplementary 1 — Sections S1 to S5 Figs. S1 to S10 Tables S1 and S2 [file research.1334.f1.pdf]

Supplementary Materials for
Single-ion anisotropy-stabilized short-period helimagnetism in frustrated chiral Co_5TeO_8

Priya R. Baral*, Ravi Yadav, Victor Ukleev, Thomas LaGrange,
Ivica Živković, Wen Hua Bi, Marek Bartkowiak, Robert Cubitt,
Nina-Juliane Steinke, Vladimir Pomjakushin, Yurii Skourski, Henrik M. Rønnow,
Oleg V. Yazyev, Arnaud Magrez, Jonathan S. White†

Email: *priya.baral@epfl.ch
Email: †jonathan.white@psi.ch

This PDF file includes:

Supplementary Text S1 to S5
Figures S1 to S10
Tables S1 and S2
Supplementary references

S1 Electronic structure and sample characterizations

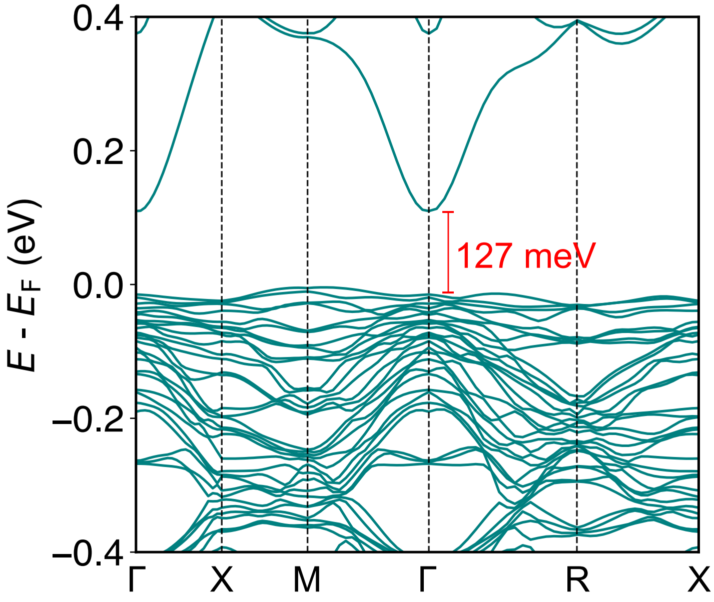

Figure S1: **Electronic structure of Co_5TeO_8.** Calculated band dispersion obtained within the LDA+U formalism, with the corresponding energy gap indicated.

Synthesis & XRD characterizations: Few single crystals from the same batch were spread over a sticky carbon tape and imaged using a scanning electron microscope (SEM). The resultant images, as shown in Fig. S2**A**, confirm Co_5TeO_8 microcrystals exhibiting octahedral morphology, with clear triangular facets, reminiscent of [111]-cubic axes. The typical size of the synthesized crystals was found to vary between 10-30 μm.

In the next step, we performed single crystal X-ray diffraction (SC-XRD) experiments on few crystallites. Unit cell parameters obtained after refinement are shown in Table S1. The resultant reciprocal space maps for l = 0 and l = 1 are shown in panel-**B** and **C** of Fig. S2. To compensate for the small crystal size, an extended data collection period of 13 hours was employed to enhance counting statistics. The appearance of forbidden reflections is attributed to anomalous Templeton scattering under this prolonged exposure. For reference, frame acquisition for ~ 10 mins, results in satisfactory structural refinement. We obtain similar goodness-of-fit while using the same structural model on different datasets obtained on multiple Co_5TeO_8 crystals.

Powder X-ray diffraction pattern was collected at room temperature. Rietveld refinement was

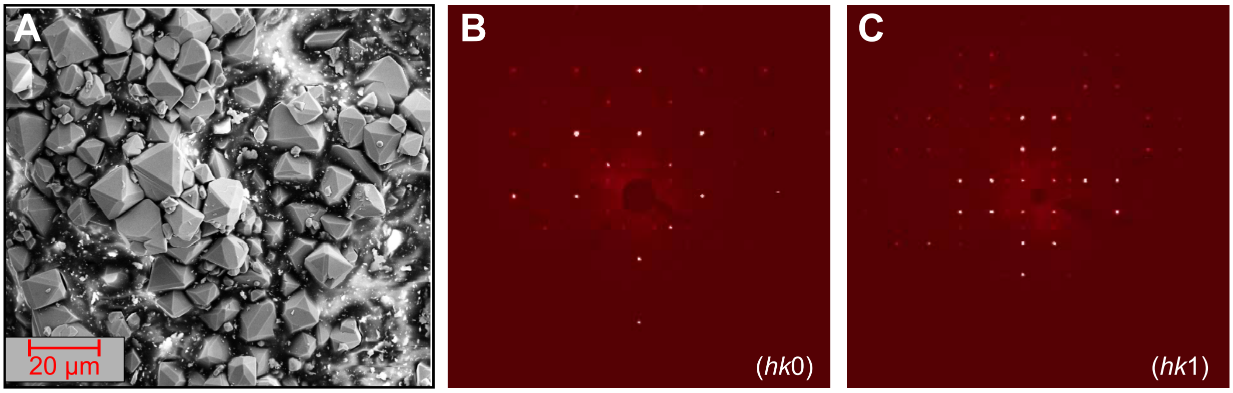

Figure S2: **Single crystal X-ray diffraction results on Co_5TeO_8.** **(A)** Shows SEM image of assembled microcrystalline Co_5TeO_8 sample. **(B)**, and **(C)** show the experimental cuts of the reciprocal space at $l = 0$ and $l = 1$ reciprocal lattice unit.

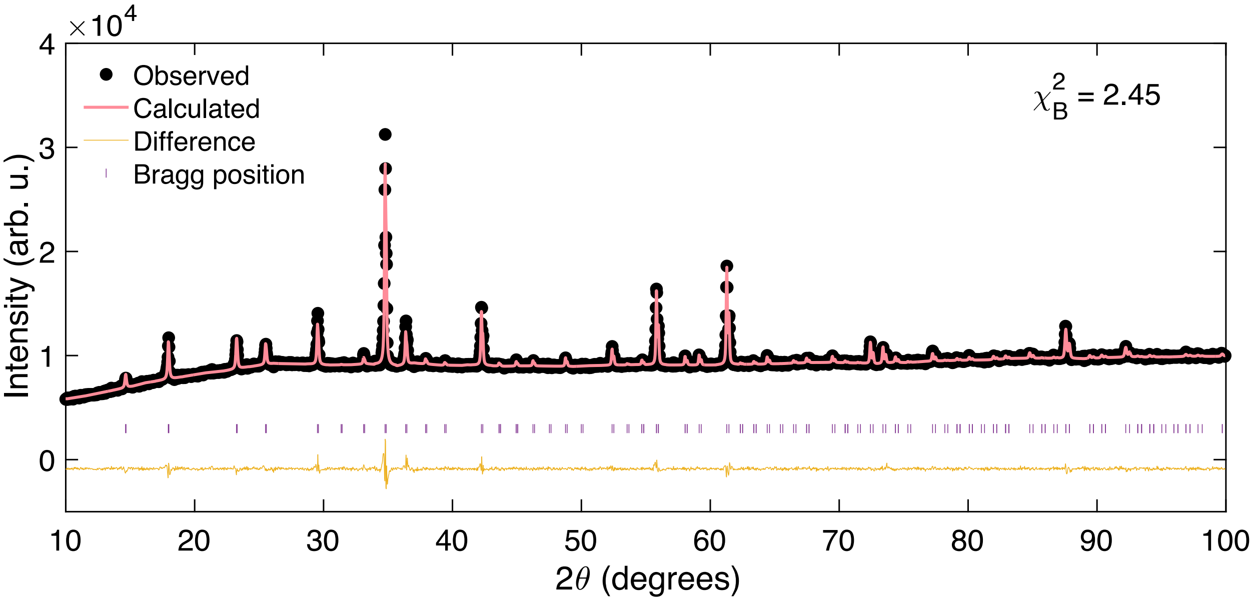

Figure S3: **Powder X-ray diffraction results on Co_5TeO_8.** Room-temperature powder X-ray diffractogram obtained on assembled polycrystalline Co_5TeO_8 sample. Results of the Rietveld refinement shows overall agreement between the lattice constants and atomic coordinates obtained using single crystal X-ray diffraction experiment.

performed on the same pattern confirming the synthesized microcrystalline sample to be pure phase Co_5TeO_8, see Fig. S3. Refined lattice parameters match with those obtained from the single crystal

structural refinement.

Table S1: Crystallographic information of Co_5TeO_8 as obtained via single crystal X-ray diffraction experiment at Mo K-edge.

Atoms	x	y	z	Occupancy	U	Wyckoff site
Co1	0.38622	0.13622	0.37500	1.00	0.016	12d
Co2	0.50027	0.50027	0.50027	1.00	0.016	8c
Te	0.12500	0.12500	0.12500	1.00	0.015	4a
O1	0.37120	0.37120	0.37120	1.00	0.017	8c
O2	0.11630	−0.10130	0.12310	1.00	0.017	24e

Electron backscatter diffraction (EBSD) analysis: Further structural and compositional analysis was carried out using electron backscatter diffraction (EBSD) and energy-dispersive X-ray spectroscopy (EDX) within a scanning electron microscope (SEM). For EBSD, silver paste was first applied to a sample stub, followed by mounting the polycrystalline Co_5TeO_8 particles and gently flattening them with a glass slide. The particles were indexed against lattice parameters imported from our CIF, and EBSD was performed on the largest grains present in the batch. Clear Kikuchi bands were obtained, allowing for unambiguous indexing. Orientation mapping identified (001), (101), and (111) facets across numerous grains. Subsequently, a focused ion beam (FIB) was used to extract a lamella from a facet oriented along [111] zone axis for the EELS analysis discussed in Section S2.

In a follow-up experiment, Co_5TeO_8 particles were dispersed in resin and cured at 180 °C for 5 minutes. To reduce electron irradiation-induced charging effects, a thin carbon coating was applied over the partially conductive resin prior to EBSD and EDX measurements on the same sample. EDX revealed that 2–4% of the crystallites exhibited complete Te deficiency. The measured atomic concentrations were: O at 53.2%, Co at 38.9%, and Te at 7.4%, slightly deviating from the nominal stoichiometry of O (57.14%), Co (35.72%), and Te (7.14%).

S2 Electron energy loss spectroscopy

The experimental determination of cobalt valence state complexes follows closely the methodologies implemented in previous studies (1,2). We adapted those approaches for scanning transmission electron microscopy (STEM) image mode in which we can characterize statistical variation in the valence states across the FIB TEM lamella with ~50 nm spatial resolution and the acquisition of ~10,000 spectra. In general, investigating oxidation states with electron microscopy and electron energy loss spectroscopy (EELS) is challenging since the electron beam irradiation at high doses can directly reduce oxides. As such, this STEM approach aim to avoid experimental artifacts from beam-induced effects and to provide statistically relevant and precise determinations of the valence states. In the following, we outline a step-by-step procedure for determining the valence states by calculating the branching ratio between the L_3 and L_2 edges of cobalt.

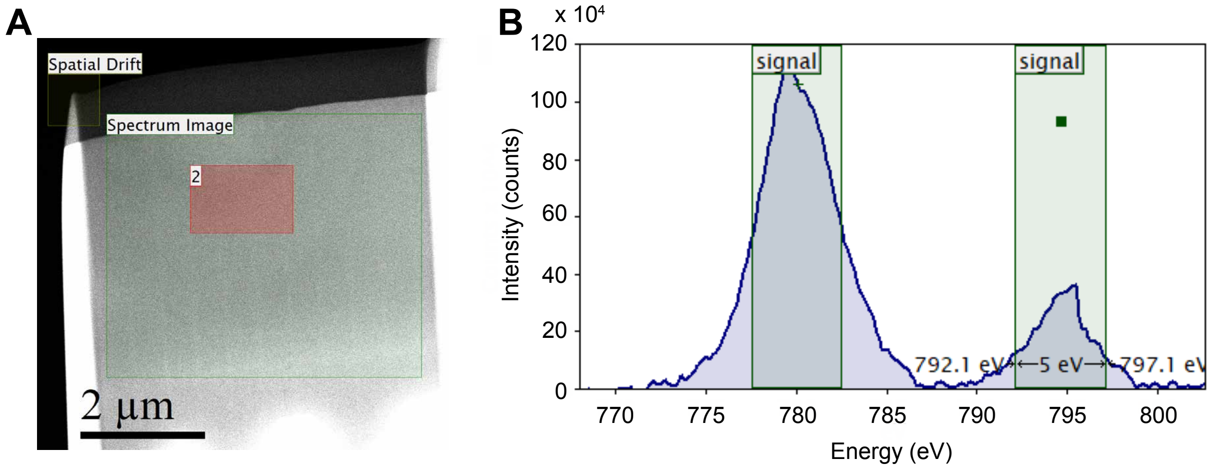

Figure S4: **Oxidation state of Co in Co_5TeO_8 determined using EELS.** **(A)** Shows the thin lamella with a uniform thickness used for scanning transmission electron microscopy (STEM) studies. The colored rectangle represents the probed with the area with electron beam. **(B)** Shows the intensity evolution across L_2 and L_3 absorption edges. The detailed procedure for spectrum analysis is outlined in Section S2. Corresponding regions for calculating the branching ratio are also highlighted.

The branching ratio is calculated by measuring the integrated counts under the M-edges and taking the ratio of $M_5/(M_4+M_5)$ or the reduced ratio L_3/L_2. This calculation is performed through a series of operations on the raw spectra in the scan region of the FIB lamella as discussed here

(see Fig. S4**A**). First, we subtract the background using the power law fitting algorithms contained within the Gatan DigitalMicrograph® software using a selection window on the pre-edge. Then, we fit the ionisiation edge of cobalt with a double arc tangent function and subtract it from the signal, leaving the peaks of L_3- and L_2-edge. We extract the integrated signal under a 5 eV wide window centered on the the peak and calculated L_3/L_2 ratio for each pixel-spectrum in the scanned regions, as shown in Fig. S4**B**. Using the extracted integral counts, we calculated the reduced L_3/L_2 over the region containing 5000 pixel-spectra, which is 4.4±0.2. The determined ratio compares well with compounds have Co^{2+} oxidation state (2,3). However, most reports state a slightly higher value of 4.8 for CoO (1). The presence of a crystal field lowers the branching ratio when it produces a low-spin ground state and the *d*-orbitals splitting in energy (crystal field theory ([Ref. 4]), which could be the scenario in Co_5TeO_8 (5).

S3 Bulk measurements

DC magnetization: These measurements were performed using either a commercial 14 T PPMS or a 7 T MPMS. Data were collected in a sweep mode, that is the system was not stabilized at specific magnetic field (or temperature) set points.

We obtain clear signature of T_{HM} as well as $T^{(1)}_{\mathrm{hyst}}$ and $T^{(2)}_{\mathrm{hyst}}$ in dc magnetization data, as shown in Fig. S5**A**. The sharp increase in $M(T)$ below $T^{(2)}_{\mathrm{hyst}}$ is indicative of a ferro(or ferri)magnetic-type ordering. By analyzing dc magnetization data deep inside the paramagnetic regime (> 150 K), we obtain a Curie-Weiss temperature of -30 K, suggestive of dominant antiferromagnetic interactions in Co_5TeO_8. This is consistent with our *ab-initio* quantum chemistry calculations as well, where we find leading-order exchange to be antiferromagnetic in nature.

Further $M(T)$ curves shown in Fig. S5**B** shows a detailed systematic evolution of all temperature-induced transitions across a wide range of applied magnetic fields. The phase boundaries thus extracted are shown in the main text Fig. 1**C**. Isothermal magnetic field scans, measured in the long-range ordered state in Co_5TeO_8 as well as above, are presented in Fig. S5**C**. We observe multiple metamagnetic-type transitions across T_{HM}, $T^{(1)}_{\mathrm{hys}}$, and $T^{(2)}_{\mathrm{hyst}}$. Phase boundaries extracted from these $M(H)$ curves have been integrated into the main text Figure 1**C**. The observed unsaturated magnetic moment forms the basis for the high-magnetic pulse field experiment, results of which

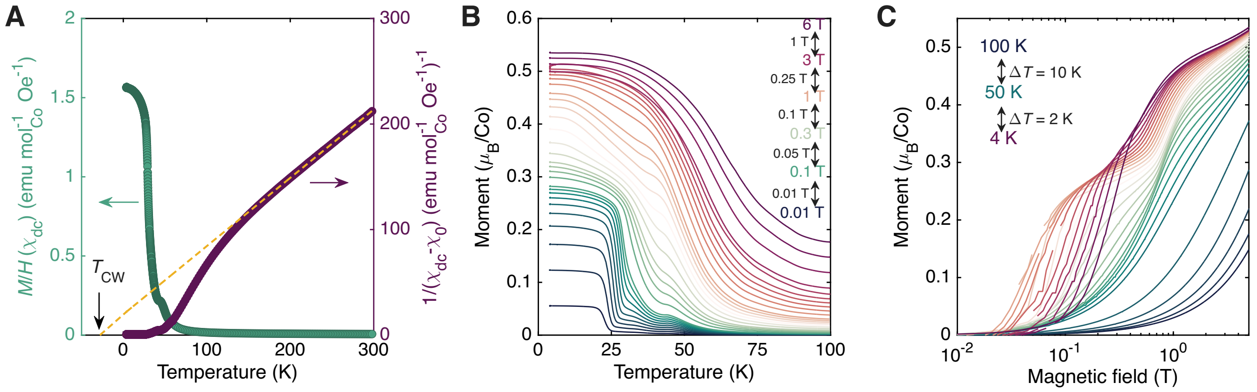

Figure S5: **Additional dc magnetization data. (A)** Dc magnetization results obtained for Co_5TeO_8 at a fixed magnetic field of 100 mT. The linear fit to the inverse magnetization data in the paramagnetic state suggests a Curie-Weiss temperature (T_{CW}) of ~-30 K. **(B)** Shows evolution of magnetic moment in Co_5TeO_8 as a function of temperature. In each case, magnetic field was kept constant at the specified value. **(C)** Magnetic isotherms measured across phase transitions in Co_5TeO_8. A wait period of 10 s was applied after stabilising at each temperature. Data were collected in the so-called "sweep-mode", that is collecting data without stabilising magnetic field at each set point.

are presented in main text Fig. 2**E**. These static magnetization measurements provides us with a high-resolution magnetic phase diagram explored using other probes, such as neutron scattering studies.

AC susceptibility: Following the static magnetization measurements, we went on to probe the phase boundaries in Co_5TeO_8 via dynamic ac susceptibility measurements. These were performed using the ACMS option of either a 7 T MPMS or the 14 T PPMS of Quantum Design (QD). Sample was enclosed tightly inside a polypropylene holder prior to being measured in either instrument. We used a small excitation field of 0.1 mT for all measurements, while the excitation frequency was kept constant at 1111 Hz. Both temperature and magnetic field were stabilized at each point prior to collecting data.

In zero field, both components of the total susceptibility, real (χ'_{ac}) and imaginary (χ''_{ac}), exhibit sharp signatures at the phase boundaries (T_{HM}, $T^{(1)}_{hyst}$, and $T^{(2)}_{hyst}$), as shown in the main text Figure 2**A**. These results are extended to further ac temperature scans, as shown in Fig. S6**A**. From our χ'_{ac} data, we find that T_{HM} is largely unaffected by increasing magnetic fields, until ~100 mT, suggesting poor renormalization of Phase-I boundary with temperature. A similar behavior was observed for

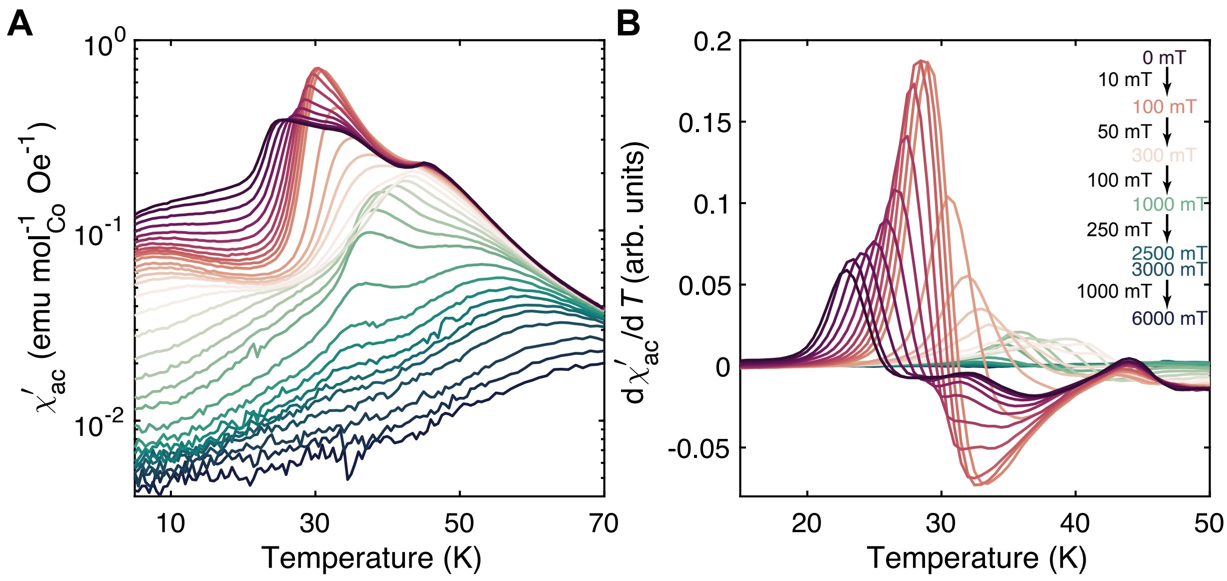

Figure S6: **Evolution of phase boundaries as probed by ac susceptibility measurements. (A)** Shows the real part of the total susceptibility, χ'_{ac}, as measured across phase transitions in Co_5TeO_8. For each scan dc magnetic field was kept constant, as indicated. **(B)** shows the derivative of the data shown in Panel-**A** with respect to temperature. For all measured χ'_{ac} data, excitation and frequency of the applied ac signal was kept constant at 0.1 mT and 1111 Hz, respectively.

the transition between Phase-I and Phase-III as well. With increasing magnetic field, the transition between Phase-II and Phase-IV is found to be sharp and almost first-order type, which can be clearly seen in the $d\chi'/dT$ data as well, see Panel-**B** of Fig. S6. With further increasing magnetic field, two broad transitions were obtained signaling the following two transitions: paramagnetic (PM) $\rightarrow$ Phase-VII $\rightarrow$ Phase-VI. These two transitions were found follow opposite behaviour, suggesting increased stability of Phase-VII against both PM as well as Phase-VI. The boundaries extracted from these measurements have been incorporated into the magnetic phase diagram shown in main text Fig. 1**C**.

Specific heat: Thermodynamics of the phase transitions observed in Co_5TeO_8 were probed using a traditional semi-adiabatic heat pulse technique inside a 14 T PPMS. A strongly pressed pellet (inset of main text Fig. 2**B**) was used for all measurements. As shown in Fig. 2**B**, we observe very weak (or no) anomalies at transition temperatures.

Thus, in order to probe their thermodynamics, we employed the so-called “long-pulse” tech-

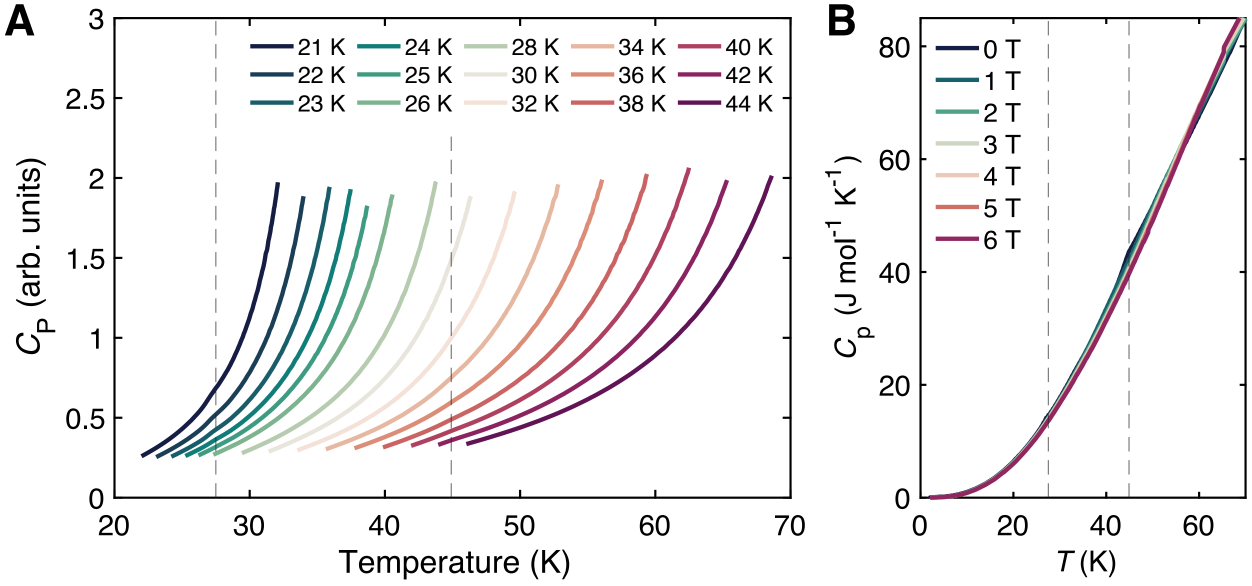

Figure S7: **Magnetic transitions probed by heat capacity measurements. (A)** Shows results of heat capacity measurements performed using the long-pulse technique at various starting temperatures, as indicated. **(B)** Shows the heat capacity as a function of temperature at fixed magnetic field. In both panels, dashed vertical lines indicate the transition temperatures, T_{HM} and $T_{\mathrm{hys}}^{(2)}$, as extracted from χ'_{ac}.

nique. Compared to the more-widely used semi-adiabatic technique where sample temperature is raised 1-2% of its initial value, here the sample is warmed up to ~70% of its initial temperature. This results in better probing of first-order-type sharp phase transitions. Figure. S7**A** summarizes the results of specific heat data of Co_5TeO_8 probed by using the "long-pulse" technique in zero magnetic field. While both methods produce weak anomalies at the phase transitions, we do not observe any significant differences among them. Applying a vertical magnetic field with respect to the pellet, we observe a diminishing transition at T_{HM}, as shown in Fig. S7**B**. Similar behaviour was previously reported for MnGe helimagnet.

Electrical capacitance measurements: To corroborate the magneto-electric (ME) behavior reported in the main text, we present additional capacitance data measured across the magnetic phase diagram of Co_5TeO_8. Near T_{HM}, $C(H)$ exhibits a weak field-dependence, appearing as a broad plateau (Fig. S8**A**). Upon cooling below T_{HM}, the normalized capacitance shows a slight increase, followed by a sharp decrease to an approximately constant value for higher fields. These features

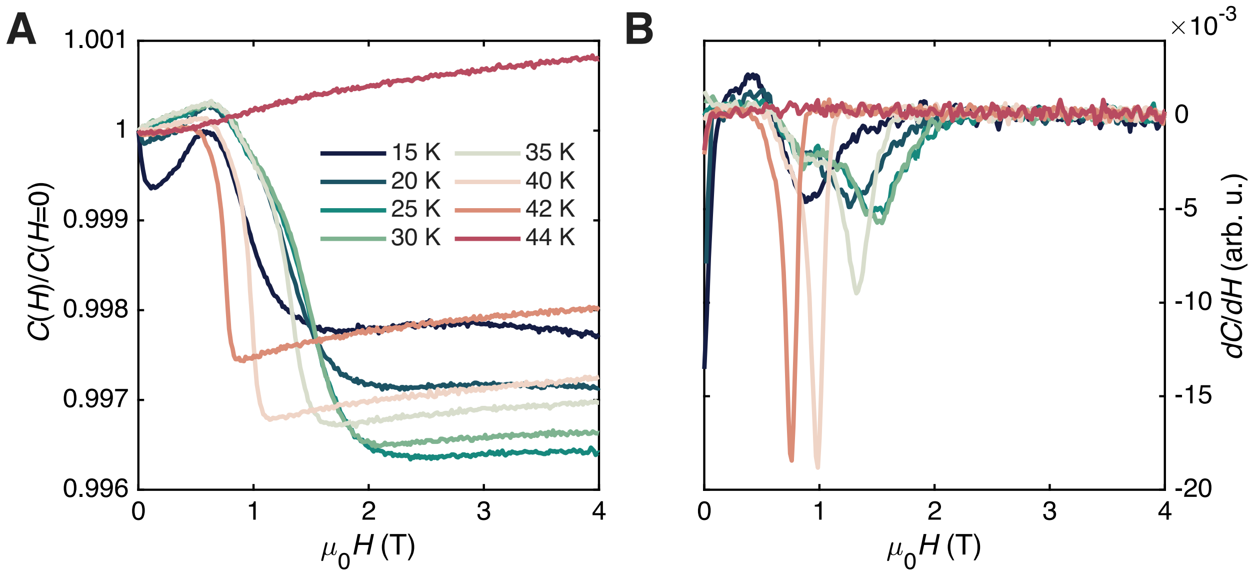

Figure S8: **Capacitance data across magnetic phase diagram. (A)** Shows the normalized capacitance data measured at various temperatures. **(B)** Shows the change in $C(H)$ with respect to magnetic field at the same temperatures.

correspond to well-defined anomalies in dC/dH, as seen in Fig. S8**B**. At the lowest measured temperature (15 K), a distinct peak emerges in $C(H)$. Together, these features align well with the magnetic phase boundaries established using other experimental probes (Fig. 1**C**), providing further evidence for macroscopic ME coupling in Co_5TeO_8 over a broad temperature and field range.

S4 Small angle neutron scattering (SANS) studies

Unpolarized SANS

Temperature dependence of $\boldsymbol{Q}_{\mathrm{IC}}$: Fig. S9**AI-IV** show temperature-dependent SANS measurements of the incommensurate magnetic structure across the hysteretic regions observed in the ac-susceptibility data. Just above T_{HM}, we observe low-$\boldsymbol{Q}$ short-range correlations that develop into long-range magnetic order upon cooling. Below T_{HM}, the propagation vector magnitude $|\boldsymbol{Q}^{(1)}|$ decreases continuously, with $\boldsymbol{Q}_{\mathrm{IC}}^{(1)}$ approaching the Γ-point ($\boldsymbol{G}_{000}$). While the first hysteretic transition at $T_{\mathrm{hys}}^{(1)}$ produces only modest changes in $|\boldsymbol{Q}^{(1)}|$ (also its intensity, as shown in Fig. S9**B**, **C**), the second transition at $T_{\mathrm{hys}}^{(2)}$ exhibits a sharp discontinuity.

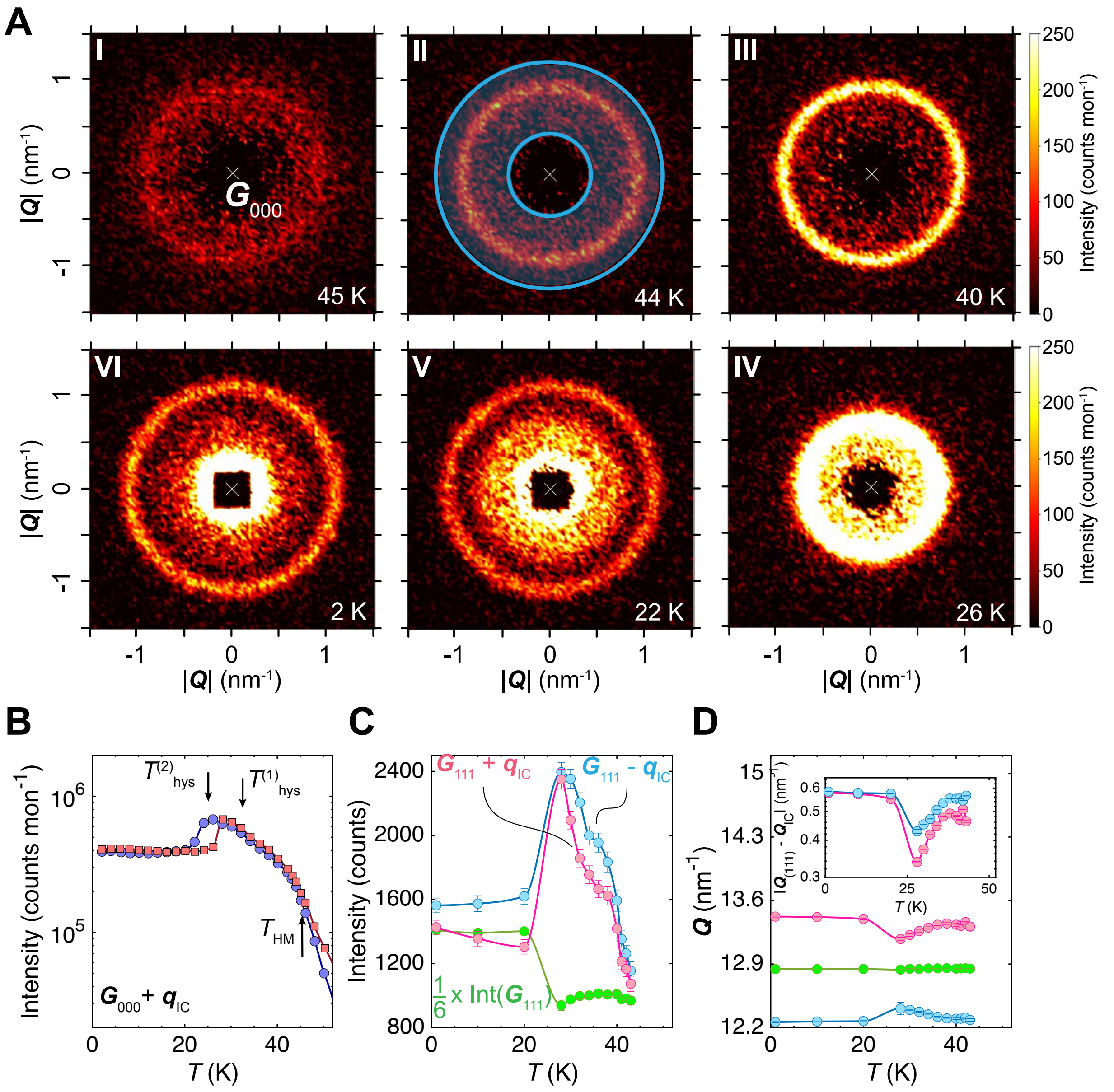

Figure S9: **Temperature-dependence of short-period incommensurate helimagnetism in zero field.** Panels-**(A) I-VI** show the SANS detector images as a function of temperature while cooling without a magnetic field (ZFC). Respective temperature is indicated within each panel. The blue ring indicated in **II** shows the sector box used for extracting intensity of the incommensurate Bragg ring as a function of temperature. For a better comparison of scattering intensity, color scale in each panel has been kept the same. *caption continues on the next page...*

Figure S9: *...caption continues from previous page.* **(B)** Shows intensity evolution across various phase transitions under ZFC condition in Co_5TeO_8 as obtained in our SANS experiment. Phase boundaries, as obtained from our χ_{ac} data, have been clearly marked by vertical arrows. Inset shows evolution of NSF-SF scattering intensity between T_{HM} and $T_{hys}^{(1)}$. **(C)** Shows the length of incommensurate magnetic wavevectors (and the corresponding pitch length of the spiral in real space, λ_h) as a function of temperature. Phase boundaries, as obtained from our χ_{ac} data, have been clearly marked by vertical arrows in Panel-**B**. **(C)** shows the intensity of second pair of incommensurate satellites tracked in our experiments, $\boldsymbol{Q}_{IC} = \boldsymbol{G}_{111} \pm \boldsymbol{q}^{(1)}$, compared with that of $\boldsymbol{G}_{111}$ nuclear reflection. **(D)** Shows the length of these incommensurate reflections $\boldsymbol{Q}_{IC}$ as a function of temperature. Inset shows the symmetric placement of each $\boldsymbol{q}^{(1)}$ around $\boldsymbol{G}_{111}$.

Thermal evolution of the helical superstructure reveals systematic wavelength variation, with the characteristic pitch length λ_h undergoing monotonic expansion from $\lambda_h(T_{HM}) = 6.96 \pm 0.02$ nm to $\lambda_h(T_{hys}^{(1)}) = 9.66 \pm 0.15$ nm, a substantial 38.8% relative elongation. This thermal evolution likely originates from the temperature variation of geometric frustration imposed by the tetrahedral framework and/or competing anisotropic exchange interactions that collectively determine the helical modulation wavelength (6). Throughout this temperature range, the helical structure maintains its chirality, as evidenced by proportional intensity increases in both NSF and SF scattering channels, see inset of Fig. S9**B**.

Below $T_{hys}^{(1)}$, the magnetic structure undergoes a distinct phase transition characterized by the disappearance of $\boldsymbol{Q}_{IC}^{(1)}$ and emergence of a new propagation vector $\boldsymbol{Q}_{IC}^{(2)}$ (see Fig. S9**AV-VI**). This new incommensurate phase exhibits $|\boldsymbol{Q}^{(2)}| = 1.1 \pm 0.1$ nm^{-1} with no detectable temperature dependence, indicating a more stable magnetic configuration. The phase transition is accompanied by enhanced intensity at $\boldsymbol{q} = 0$ reflections observed by PND, as shown in Fig. S9**D-E**, suggesting the development of an additional commensurate magnetic component and change in magnetic domain populations.

Polarized SANS

Following the comprehensive unpolarized neutron diffraction investigation, we proceeded to conduct polarization-analysis measurements utilizing the small-angle neutron scattering (PolSANS)

configuration at the D33 instrument of Institut Laue Langevin (ILL) in Grenoble. We implemented the longitudinal geometry, wherein the incident neutron beam remains parallel to the applied magnetic field. Throughout the measurements, a weak longitudinal magnetic field (50 mT) was applied to the sample. Additionally, a 10 mT guide field was maintained to lock the neutron spin quantization axis, which was oriented along the incident neutron beam direction. The scattered neutron beam analysis was performed using a spin-polarized ^{3}He cell, with a maximum anticipated polarization of 98.6%.

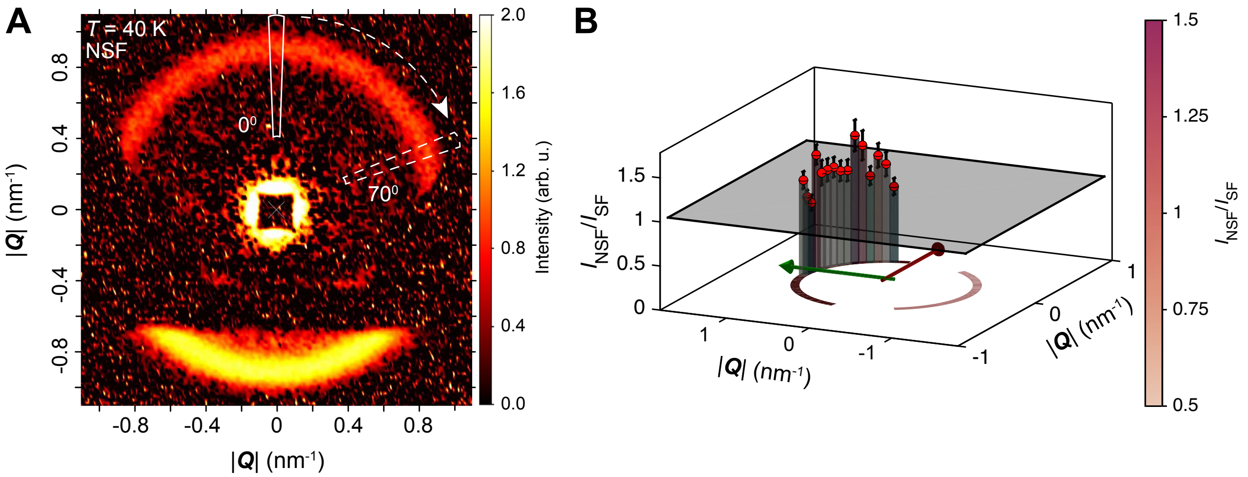

Figure S10: **Comprehensive analysis of non-spin-flip (NSF) and spin-flip (SF) scattering from polarized neutron scattering measurements.** **(A)** Displays the resultant detector image at T = 40 K from the NSF scattering process. Note that the upper portion of this detector image is presented in main text Fig. 3**F**. The asymmetric intensity distribution between the upper and lower arcs arises from the presence of the ^{3}He analyzer positioned in front of the former arc. One-dimensional radial $\boldsymbol{Q}$-scans were extracted using the white sector boxes delineated on the map. Each line scan was fitted with a Lorentzian function to determine the integrated intensity. **(B)** Histograms display the ratio between NSF scattering intensity and SF scattering intensity from the sector boxes illustrated in Panel-**(A)**, with their corresponding error bars. The thick red arcs denotes the magnetic scattering intensity on the detector. The dark solid rectangle represents a reference plane where $I_{\mathrm{NSF}} = I_{\mathrm{SF}}$.

Owing to the relatively large momentum transfer (~1 nm^{-1}, see main text Fig. 3**B**), the ^{3}He cell covered only the upper portion of the circular SANS pattern (Fig. S10**A**), producing an order-of-magnitude difference in scattered intensity between the upper and lower arcs. The upper arc,

shown in Fig. 3**F** of the main text, was examined in greater detail here by analyzing non–spin-flip (NSF) and spin-flip (SF) intensities. We used narrower sector boxes, spanning 0–70° in 5° azimuthal increments, as indicated schematically in Fig. S10**A**. Even with this finer selection, the ratio $I_{\mathrm{NSF}}/I_{\mathrm{SF}} = 1.09 \pm 0.18$ was obtained. This near-equal distribution of NSF and SF scattering is consistent with a Bloch-type spin modulation, in agreement with the conclusions of the main text.

S5 Cluster-based quantum chemistry calculations

Point charge construction on the local manifold

For quantum chemistry calculations, a cluster was chosen that comprises a central unit containing two Co edge sharing polyhedra [Co_2O_x] (x = 10 or 8) for exchange parameter calculations and nearest neighbor (NN) Co polyhedra. The polyhedra adjacent to central unit were explicitly included in the quantum chemistry computations to describe the finite charge distribution in the immediate neighborhood, while the remaining part of the extended solid-state matrix was modeled as a finite array of point charges fitted to reproduce the ionic Madelung field in the cluster region (7). This procedure ensures that the quantum cluster experiences an electrostatic environment representative of the bulk crystal while maintaining overall charge neutrality. The Co ions of the central unit were modeled using all electron basis functions of quadruple-zeta quality (8), while all-electron basis sets of quintuple-zeta quality (9) were employed for the bridging ligands in the central unit. The remaining O ligands in the central unit were modeled with all-electron basis functions of triple-zeta quality (9). Co sites of polyhedra adjacent to the reference unit were described using all electron basis functions of triple-zeta quality. Ligands of these adjacent polyhedra were modeled with all electron double-zeta basis functions. Te ions in the cluster were described using energy-consistent relativistic pseudopotentials along with a single s-basis function (10).

All quantum chemistry computations were performed with the MOLPRO quantum chemistry package (11). Electron correlations were captured using complete-active space self-consistent field (CASSCF) level of theory. Active space in CASSCF calculations included six t_{2g} orbitals at the two Co sites (in the central unit). In the subsequent multi-reference configuration interaction (MRCI) (12,13) the Co t_{2g} orbitals along with O $2p$ orbitals of the bridging ligand were included

in the active space. The MRCI calculations involved single and double excitations from the t_{2g} shells of the Co ions and the p valence shells of the bridging ligands. The Pipek-Mezey localization module (14) available in MOLPRO was employed for separating the Co-3d and O-2p valence orbitals into different groups, i.e., centered at sites of either the central unit or at sites of the NN octahedra. The spin-orbit treatment was carried out at each level of theory as described by A. Berning *et al.* (Ref.15).

Intra- and inter-site exchanges

We determined the intra-site interactions appearing in $\mathcal{H}^{\mathrm{II}}$, i.e., the single-ion anisotropy $\mathcal{A}$, using the one-site model. The single-ion anisotropy quantifies the zero-field splitting of the ground state that stems from spin-orbit and crystal-field effects. This quantity was derived based on the methodology developed in Ref. (16), taking advantage of the multiplet structure and corresponding wavefunctions. In brief, the mixing of the low-lying $^4T_1$ states with the higher-lying states was treated perturbatively and the spin-orbit wavefunctions related to the high-spin configuration were projected onto the space spanned by the $^4T_1$ $|\mathcal{S}, M_f\rangle$ states. The ortho-normalized projections of the low-lying quartet wavefunctions, $\tilde{\psi}_k$, and the corresponding eigenvalues, E_k, were used to construct the effective Hamiltonian $\tilde{\mathcal{H}}_{\mathrm{eff}} = \Sigma_k E_k |\tilde{\psi}_k\rangle\langle\tilde{\psi}_k|$. A one-to-one correspondence between $\tilde{\mathcal{H}}_{\mathrm{eff}}$ and the model Hamiltonian $\tilde{H}_{\mathrm{mod}} = \mathcal{S} \cdot \bar{\bar{\mathcal{A}}} \cdot \mathcal{S}$ led to the $\bar{\bar{\mathcal{A}}}$ tensor, which was then diagonalized to obtain the axial parameter, $\mathcal{A}$.

Table S2: **Local exchange parameters obtained via quantum chemistry calculations.** Microscopic exchange parameters for Co_5TeO_8, as obtained from our many-body wavefunction-based quantum chemistry calculations. Atomic manifolds are as schematically illustrated in Fig. 1**A**. All output exchange parameters are expressed in units of meV.

Co-sites	r (Å)	$\mathcal{J}_{ij}$	$\mathcal{K}_{ij}$	$\mathcal{D}_x$	$\mathcal{D}_y$	$\mathcal{D}_z$	$\lvert\mathcal{D}/\mathcal{J}\rvert$
Co_1-Co_1	3.69	0.116	−0.0084	−0.011	0.073	0.0656	0.84
Co_2-Co_2	2.95	0.088	−0.0011	0.002	0.003	0.001	0.03
Co_1-Co_2	3.65	0.208	−0.0032	0.003	0.001	0.021	0.102

Furthermore, we derived the inter-site interactions appearing in the spin Hamiltonian $\mathcal{H}^{\mathrm{I}}$, i.e.,

the bilinear and biquadratic isotropic exchange couplings $\mathcal{J}_{ij}$ and $\mathcal{K}_{ij}$, respectively, along with the antisymmetric anisotropic vector, $\mathcal{D}$. We relied on the two-site model shown in Figure 1**A**. The resulting *ab initio* Hamiltonian was mapped onto the anisotropic biquadratic model Hamiltonian given in Equation 3, which involved sixteen spin-orbit states corresponding to one septet, one quintet, one triplet, and a singlet. The mapping was accomplished using the procedure described in Ref. [17]. The resulting magnetic interactions are listed in Table S2. The dominant term among the inter-site interactions is the bilinear isotropic exchange coupling $\mathcal{J}_{ij}$, the positive sign of which indicates an anti-parallel spin interaction between the S=3/2 centers. We find that all leading order bilinear Heisenberg exchanges are AFM in nature, whereas biquadratic terms are always FM.

To predict helimagnetic transition temperature in Co_5TeO_8, we combined calculated exchange interactions with a functional form incorporating SIA contributions (18,19):

$$T_C = \frac{1}{3k_B}\left[(z_1J_1 + z_2J_2)S(S+1) + D\{2S(S+1) - 1\}\right]. \tag{S1}$$

Using the exchange parameters listed in Table S2, we estimated a T_{HM} of 40.34 K, which agrees well with the experimentally observed value of 44.9 K. This agreement between theoretical predictions and experimental measurements provides an internal consistency check for the proposed interaction hierachy and indicates that the calculated energy scales are of the correct order of magnitude. It should not, however, be taken as a unique validation of the full microscopic Hamiltonian.

Supplementary References

S[1] C. S. Bonifacio, S. Carenco, C. H. Wu, S. D. House, H. Bluhm, and J. C. Yang, Thermal stability of core-shell nanoparticles: A combined in situ study by XPS and TEM, https://pubs.acs.org/doi/10.1021/acs.chemmater.5b01862Chemistry of Materials **27**, 6960 (2015).

S[2] M. Koshino, H. Kurata, S. Isoda, and T. Kobayashi, Branching ratio and $L_2 + L_3$ intensities of $3d$-transition metals in phthalocyanines and the amine complexes, https://doi.org/10.1016/S0968-4328(99)00116-XMicron **31**, 373 (2000).

S[3] Z. Wang, J. Yin, and Y. Jiang, EELS analysis of cation valence states and oxygen vacancies in magnetic oxides, https://doi.org/10.1016/S0968-4328(99)00139-0Micron **31**, 571 (2000).

S[4] H. Tan, J. Verbeeck, A. Abakumov, and G. Van Tendeloo, Oxidation state and chemical shift investigation in transition metal oxides by EELS, https://doi.org/10.1016/j.ultramic.2012.03.002Ultramicrosc [illegible] **116**, 24 (2012).

S[5] S. Podchezertsev, N. Barrier, A. Pautrat, E. Suard, M. Retuerto, J. A. Alonso, M. T. Fernández-Díaz, and J. Rodríguez-Carvajal, Influence of polymorphism on the magnetic properties of Co_5TeO_8 spinel, https://pubs.acs.org/doi/10.1021/acs.inorgchem.1c01131Inorganic Chemistry **60**, 13990 (2021).

S[6] P. R. Baral, O. I. Utesov, C. Luo, F. Radu, A. Magrez, J. S. White, and V. Ukleev, Direct observation of exchange anisotropy in the helimagnetic insulator Cu_2OSeO_3, https://doi.org/10.1103/PhysRevRese [illegible] Physical Review Research **5**, L032019 (2023).

S[7] M. Klintenberg, S. E. Derenzo, and M.J. Weber, Accurate crystal fields for embedded cluster calculations, https://doi.org/10.1016/S0010-4655(00)00071-0Computer Physics Communications **131**, 120 (2000).

S[8] N. B. Balabanov, and K. A. Peterson, Systematically convergent basis sets for transition metals. I. All-electron correlation consistent basis sets for the $3d$ elements Sc–Zn, https://doi.org/10.1063/1.1998907The Journal of Chemical Physics **123**, 064107 (2005).

S[9] T. H. Dunning, Jr., Gaussian basis sets for use in correlated molecular calculations. I. The

atoms boron through neon and hydrogen, https://doi.org/10.1063/1.456153Journal of Chemical Physics **90**, 1007 (1989).

S[10] H. Stoll, B. Metz, and M. Dolg, Relativistic energy-consistent pseudopotentials—Recent developments, https://doi.org/10.1002/jcc.10037Journal of Computational Chemistry **23**, 767 (2002).

S[11] H.-J. Werner, P. J. Knowles, G. Knizia, F. R. Manby, and M. Schütz, Molpro: a general-purpose quantum chemistry program package, https://doi.org/10.1002/wcms.82Wiley Interdisciplinary Reviews: Computational Molecular Science **2**, 242 (2012).

S[12] H.-J. Werner, and P. J. Knowles, An efficient internally contracted multiconfiguration–reference configuration interaction method, https://doi.org/10.1063/1.455556The Journal of Chemical Physics **89**, 5803-5814 (1988).

S[13] P. J. Knowles, and H.-J. Werner, Internally contracted multiconfiguration-reference configuration interaction calculations for excited states, https://doi.org/10.1007/BF01117405Theoretica chimica acta **84**, 95-103 (1992).

S[14] J. Pipek, and P. G. Mezey, A fast intrinsic localization procedure applicable for *ab initio* and semiempirical linear combination of atomic orbital wave functions, https://doi.org/10.1063/1.456588The Journal of Chemical Physics **90**, 4916–4926 (1989).

S[15] A. Berning, M. Schweizer, H.-J. Werner, P. J. Knowles, and P. Palmieri, Spin-orbit matrix elements for internally contracted multireference configuration interaction wavefunctions, https://doi.org/10.1080/00268970009483386Molecular Physics **98**, 1823–1833 (2000).

S[16] R. Maurice, R. Bastardis, C. d. Graaf, N. Suaud, T. Mallah, and N. Guihery, Universal theoretical approach to extract anisotropic spin Hamiltonians, https://pubs.acs.org/doi/10.1021/ct900326eJournal of Chemical Theory and Computation **5**, 2977 (2009).

S[17] N. A. Bogdanov, R. Maurice, I. Rousochatzakis, J. van den Brink, and L. Hozoi, Magnetic state of pyrochlore $Cd_2Os_2O_7$ emerging from strong competition of ligand distortions and longer-range crystalline anisotropy, https://link.aps.org/doi/10.1103/PhysRevLett.110.127206Physical Review Letters **110**, 127206 (2013).

S[18] S. K. Choi, R. Coldea, A. N. Kolmogorov, T. Lancaster, I. I. Mazin, S. J. Blundell, P. G. Radaelli, Y. Singh, P. Gegenwart, K. R. Choi, S.-W. Cheong, P. J. Baker, C. Stock, and J. Taylor, Spin waves and revised crystal structure of honeycomb iridate Na_2IrO_3, https://link.aps.org/doi/10.1103/PhysRevLett.108.127204Physical Review Letters **108**, 127204 (2012).

S[19] V. M. Katukuri, S. Nishimoto, V. Yushankhai, A. Stoyanova, H. Kandpal, S. Choi, R. Coldea, I. Rousochatzakis, L. Hozoi, and J. Van Den Brink, Kitaev interactions between $j = 1/2$ moments in honeycomb Na_2IrO_3 are large and ferromagnetic: insights from *ab initio* quantum chemistry calculations, https://iopscience.iop.org/article/10.1088/1367-2630/16/1/013056/metaNew Journal of Physics **16**, 013056 (2014).
